# Mesenchymal stem cell transplantation ameliorates Sjögren’s syndrome via suppressing IL-12 production by dendritic cells

**DOI:** 10.1186/s13287-018-1023-x

**Published:** 2018-11-08

**Authors:** Bingyu Shi, Jingjing Qi, Genhong Yao, Ruihai Feng, Zhuoya Zhang, Dandan Wang, Chen Chen, Xiaojun Tang, Liwei Lu, Wanjun Chen, Lingyun Sun

**Affiliations:** 1 Department of Rheumatology and Immunology, The Affiliated Drum Tower Hospital of Nanjing University Medical School, Nanjing, China; 2 Department of Pathology and Center of Infection and Immunology, University of Hong Kong, Hong Kong, China; 3 National Institute of Dental and Craniofacial Research, NIH, Bethesda, MD USA

**Keywords:** Mesenchymal stem cells, Sjögren’s syndrome, Interleukin-12

## Abstract

**Background:**

Mesenchymal stem cells (MSCs) have been demonstrated to be effective in treating autoimmune diseases including Sjögren’s syndrome (SS). We aim to compare the effects of MSC transplantation (MSCT) and the role of serum interleukin-12 (IL-12) in SS.

**Methods:**

IL-12 levels were measured by ELISA. IL-12 mRNA transcripts in dendritic cells (DCs) were determined by RT-PCR. After co-culturing with MSCs, IL-12 mRNA transcripts in mouse and human DCs were detected. Non-obese diabetic (NOD) mice received MSCT, recombinant IL-12, or anti-IL-12 mAb treatment, respectively. Then, salivary flow rates, histopathology of salivary glands, and splenic lymphocyte subsets were examined in these mice.

**Results:**

IL-12 levels in the serum were significantly increased in SS patients and positively correlated with the EULAR 2010 Sjögren’s syndrome disease activity index. DCs from SS patients produced more IL-12 than those from the control. Likewise, IL-12 treatment in NOD mice significantly decreased salivary flow rates and promoted lymphocyte infiltration in salivary glands. IL-12 antibodies downregulated Th1, Th17, and Tfh cell. MSCT enhanced salivary flow rates and decreased lymphocyte infiltrations in salivary glands of NOD mice. MSCT downregulated Th17 and Tfh cells but upregulated regulatory T cells. MSCT reduced IL-12 productions in both SS patients and mice.

**Conclusion:**

Our results indicate that MSCs ameliorate SS possibly via suppressing IL-12 production in DCs and that IL-12 could be a potential therapeutic target of SS.

**Trial registration:**

NTC00953485. Registered June 2009.

**Electronic supplementary material:**

The online version of this article (10.1186/s13287-018-1023-x) contains supplementary material, which is available to authorized users.

## Background

Mesenchymal stem cells (MSCs), derived from either the bone marrow (BMMSCs), umbilical cord (UCMSCs), or adipose tissues (ADMSCs), have been shown to be effective in treating various autoimmune diseases, including systemic lupus erythematosis (SLE), rheumatoid arthritis (RA), and inflammatory bowel disease (IBD) [1–4]. However, much less is known about the effects of MSCs in treating Sjögren’s syndrome (SS). In mice with radiation-induced xerostomia, MSC transplantation (MSCT) has been found to reduce apoptotic cell rates in salivary glands (SG) and increase functional acini, thus ameliorating salivary damages [5, 6]. Several studies have reported the use of BMMSCs in treating SS. Khalili et al. reported that BMMSCs could prevent saliva secretion loss and reduce lymphocytic influx in salivary glands in non-obese diabetic (NOD) mice [7]. Our previous studies show that SS patients with poor response to glucocorticoid or glucocorticoid combined with immunosuppressive drugs could also benefit from UCMSC transplantation [8]. These data provide strong evidences for the advantages of MSCT application in treating SS patients. However, the mechanisms underlying the beneficial effects of MSCs on SS are not fully elucidated.

IL-12 is a pro-inflammatory cytokine mostly produced by dendritic cells (DCs) [9]. IL-12 acts as a pro-inflammatory cytokine because of its capacity to induce T-bet and promote naïve T cell differentiation into Th1 cells. It was reported that overexpression of IL-12 in submandibular gland of autoimmune-prone SJL mice contributed to the manifestation of SS-like symptoms and that the mice displayed age-dependent increase in anti-SSB/La and anti-nuclear antibodies [10], which suggested IL-12 might accelerate the pathogenesis of SS. However, the mechanism of IL-12 on SS remains incompletely understood. Apart from inflammatory infiltrates in salivary glands, Sjögren’s syndrome is also a disease with lymphocytic proliferation disorders. For example, it has been reported that Th1, Th17, and Tfh cells were highly elevated in SS patients, while Th2 and Treg cells were decreased [11–14]. The NOD mice displayed similar lymphocyte alterations to SS patients [8]. Thus, we aimed to study the effects and mechanisms of IL-12 on SS focusing on the local infiltrates and changes of lymphocyte subsets.

MSCs possess strong immuno-regulatory functions [15]. In addition to regulating T and B cells, MSCs are also capable of regulating DCs [16–19]. It was reported that MSCs strongly inhibited the differentiation of peripheral monocytes into DCs via prostaglandin E2 (PGE2) [20]. Moreover, MSCs could also downregulate the expression of activation markers such as MHC-II, CD86, and CD40 on DCs [21]. Co-cultures with MSCs and DCs increased the generation of IL-6, IL-10, and CXCL8 but decreased TNF-α, IFN-γ, and CXCL10 in DCs [16]. However, how MSCs regulate the generation and function of DCs in SS remains unknown.

In this study, we aimed to explore the effects of MSCT and the role of IL-12 in SS.

## Methods

### Isolation and culture of UCMSCs and fibroblast-like synoviocytes (FLS)

Fresh umbilical cords of newborn babies and synovial tissues from human knee joints were obtained from The Affiliated Drum Tower Hospital and were processed immediately. Protocols for isolation and culture of UCMSCs and FLS were described previously [22, 23]. Passages 3–5 UCMSCs and FLS were used in this study. The protocols were approved by the Ethics Committee at the Drum Tower Hospital of Nanjing University Medical School.

### Mice

Female NOD(ltj) mice at 8–12 weeks of age obtained from the Model Animal Research Center of Nanjing University were used for studying experimental Sjögren’s syndrome. All animal experiments were approved by the Ethics Committee of the Affiliated Drum Tower Hospital of Nanjing University Medical School. The 8-week-old mice were injected with one million human UCMSCs, FLS, or the same volume of sterilized phosphate-buffered saline (PBS) via tail vein, respectively. In separate experiments, 12-week-old NOD mice were injected intraperitoneally (i.p) with murine recombinant IL-12 proteins (40 ng/g body weight, IL-12 group) or anti-IL-12 monoclonal antibody (4 μg/g body weight, anti-IL-12 group) or same volume of sterilized PBS. Five mice were used for each group. All mice were sacrificed at 13 weeks of age.

### Patients and healthy controls

Twenty-nine SS patients and age- and sex-matched healthy controls (HC) were studied. All SS patients fulfilled the American–European Consensus Group criteria for primary SS diagnosis [24]. We scored the disease activity index of each patient according to the EULAR 2010 Sjögren’s syndrome disease activity index (ESSDAI) criteria [25]. For comparing serum IL-12 levels before and after MSC transplantation, 10 SS patients were studied. UCMSCs (1 × 10^6^/kg of body weight) were administrated by intravenous infusion. Whole blood was taken before and 7 days post MSCT. This protocol was approved by the Ethics Committee at the Drum Tower Hospital of Nanjing University Medical School and registered at http://www.clinicaltrials.gov (NTC00953485). All patients provided informed consent. Detailed clinical features about SS patients who received MSCT were listed in Additional file 1: Table S1.

### Measurement of saliva flow

The saliva flow rates were measured as previously described [26]. Briefly, mice were anesthetized by i.p injection with 10% chloral hydrate at a dose of 100 ml/kg body weight. After 5 min, anesthetized mice were induced for saliva secretion by i.p injection of 5 mg/kg pilocarpine (Sigma-Aldrich), and then a 20-μl sized pipet tip was used to collect saliva from the oral cavity for 10 min at room temperature.

### Flow cytometry analysis

For detection of mouse Th1, Th2, and Th17 cells and expression of IL-12 in human DCs, mouse splenocytes or human PBMCs were first stimulated with phorbol 12-myristate 13-acetate (PMA) (50 ng/ml), ionomycin (1 μg/ml), and brefeldin A (5 μg/ml) (Enzo, Lörrach, Germany) at 37 °C for 5 h. After stimulation, cells were washed with PBS. Mouse cells were then stained with anti-mouse CD4 for 30 min on ice, washed with PBS, and finally stained with intracellular cytokines (anti-mouse IFN-γ, IL-4, or IL-17A) for 30 min using a Fixation/Permeabilization Kit (Nordic-MUbio, Susteren, The Netherlands). Human cells were then stained with anti-human linage cocktail and anti-human HLA-DR for 30 min on ice, washed with PBS, and stained with anti-human IL-12p40 using the same Fixation/Permeabilization Kit. Unstimulated mouse splenocytes were stained for detection of Tfh and Treg cells. For detection of Treg cells, cells were first stained with anti-mouse CD4 for 30 min on ice, washed with PBS, permeated by a Foxp3 staining buffer (ebioscience, San Diego, CA) for 1 h, washed with PBS, and stained with anti-mouse Foxp3 for 30 min. For staining of Tfh cells, splenocytes were stained with anti-mouse CD4, CXCR5, and PD-1 for 30 min on ice. Data were acquired using a FACS calibur system (BD Biosciences, San Jose, CA) and analyzed by FlowJo software. All antibodies were purchased from Miltenyi Biotec (Bergisch Gladbach, Germany) or eBioscience (San Diego, CA, USA).

### Cytokine detection

After collection of whole blood from SS patients and healthy controls, samples were immediately centrifuged at 1400*g* for isolation of serum; serum was then subpackaged and stored at − 80 °C to avoid repeated freeze/thaw cycles. All samples were brought to room temperature before cytokine detection. Levels of serum IL-12 in SS patients and healthy controls were detected by enzyme-linked immunosorbent assay (ELISA) (R&D systems, D1200). The experiments were performed according to the manufacturer’s instructions. For measurement of IL-12 levels in SS patients or NOD mice before and after MSCT, luminex chips assay (Merck&Millipore, MA, USA) was used.

### Human and mouse DC preparation

For generating human monocyte-derived DCs, peripheral blood mononuclear cells (PBMCs) were isolated from healthy subjects by Ficoll-Paque density gradient centrifugation. CD14+ monocytes were isolated by magnetic cell sorting kit (Miltenyi, 130-097-052) according to the manufacturer’s instructions. Purified CD14+ cells were cultured in 24-well plate in complete RPMI-1640 media and stimulated with 100 ng/ml granulocyte–macrophage colony-stimulating factor (GM-CSF) plus 100 ng/ml IL-4 for 5 days for induction of immature DCs. Subsequently, 100 ng/ml lipopolysaccharides (LPS) was added to induce DC maturation. Forty-eight hours later, the cells were used as human monocyte-derived DCs. CD11c+ cells were isolated from splenocytes by magnetic cell sorting kit (Miltenyi, 130-097,059) and used as mouse DCs.

### UCMSC-DC co-culture experiments

DCs were prepared as described above. We used monocyte-derived DCs generated from HC subjects and CD11c+ DCs from C57BL/6 mice in the co-culture experiments. A trans-well system (Corning, Corning, NY, USA) was used to perform the co-culture experiments. DCs were plated in the lower chamber. UCMSCs of passages 3–5 were seeded into the trans-well membrane of the inner chamber with 0.4-μm pore size prior to the co-culture experiment to allow adherence overnight; cells were cultivated in complete RPMI 1640 medium. The ratio of UCMSCs to DCs was 1:5. Forty-eight hours after co-culture, cells were harvested for conducting further experiments.

### RNA isolation and real-time polymerase chain reaction (RT-PCR)

Total RNA samples were extracted from human or mouse DCs. Complementary DNA (cDNA) was synthesized by PrimeScript RT regent kit (Takara Biotechnology, Tokyo, Japan). Real-time PCR was performed to detect IL-12 mRNA levels on an Applied Biosystems 7500 real-time PCR system (Applied Biosystems, Foster City, USA). Data analysis was performed using an SDS software (version 2.0, Applied Biosystems). Primers were designed and synthesized by Takara Biotechnology. The relative expressions of each gene were determined and normalized to the expression of housekeeping gene glyceraldehyde 3-phosphate dehydrogenase (GAPDH) and calculated using the 2^−ΔΔCT^ method. Primer sequences were listed in Additional file 1: Table S2.

### Histologic analysis

After mice were sacrificed, submandibular glands (SGs) were collected and immediately fixed in 4% paraformaldehyde. Paraformaldehyde-fixed tissues were embedded in paraffin. Serial 4-μm sections were cut and stained with hematoxylin and eosin (H&E) for morphologic examination. Chisholm–Mason classification criteria were applied to define lymphocyte infiltrations in SGs [27].

### Statistical analysis

Data was presented as mean ± SD in each group. All statistical analyses were performed using GraphPad Prism 6 software (GraphPad Software, La Jolla, CA, USA). Spearman’s test was carried out to analyze correlations of IL-12 levels with ESSDAI scores. Statistical significances were determined by one-way ANOVA. When only two groups were analyzed, paired or unpaired Student *t* test was used. A *p* value < 0.05 was considered significant difference.

## Results

### Increased IL-12 in SS patients positively correlates with disease activity

**Fig. 1:**
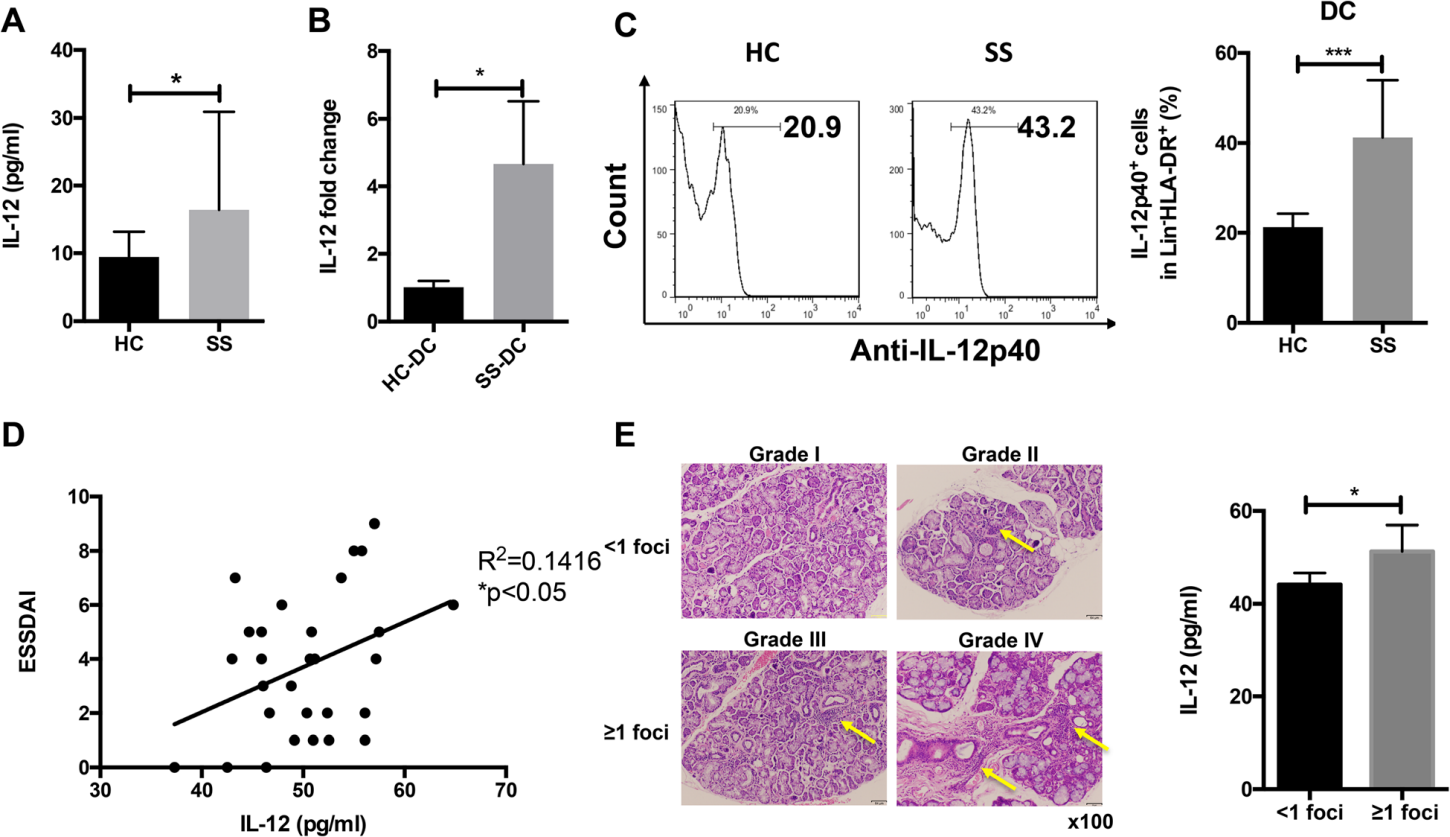
Elevated IL-12 levels in SS patients. **a** Serum IL-12 levels of SS patients and healthy controls (*n* = 29 for each group). **b** IL-12 mRNA expression in monocyte-derived DCs (*n* = 5 for each group). **c** IL-12p40 expression in Lin-HLA-DR+ subgroups from SS/HC PBMCs (*n* = 12 for each group). **d** Correlation analysis of IL-12 levels and ESSDAI scores (*n* = 29). **e** Serum IL-12 levels in SS patients. Error bars denote ± SD.*p < 0.05, ***p < 0.001

To explore the differential expression levels of IL-12 in SS patients and HC subjects, we measured serum IL-12 levels by ELISA. Figure 1a shows that serum IL-12 levels were significantly increased in SS patients (*p* < 0.05). As dendritic cells were the main producer of IL-12 in vivo, we then detected IL-12 expression in DCs. Figure 1b shows that the in vitro generated monocyte-derived DCs from SS patients expressed higher IL-12 mRNA levels (*p* < 0.05). Besides, flow cytometry analysis also showed higher IL-12 expression levels in Lin-HLA-DR+ subgroups (which represent DCs) in SS VS HC PBMCs (*p* < 0.001). This data suggested that elevated IL-12 might be related to SS pathogenesis. In order to detect the possible relationship, we scored the ESSDAI of SS patients and performed correlation analysis of ESSDAI scores with IL-12 levels by Spearman’s test. Figure 1d shows that IL-12 levels positively correlated with ESSDAI scores (*p* < 0.05, *R*^2^ = 0.1416). Lymphocyte infiltrations in SGs of SS patients were determined by Chisholm–Mason grade. Of 29 SG biopsy slides, 4 had a Chisholm–Mason grade < I and 25 had a grade ≥ I, and serum IL-12 levels were significantly higher in the latter group (*p* < 0.05, Fig. 1e), suggesting that IL-12 might accelerate lymphocyte infiltrations into SGs in SS patients. However, IL-12 levels did not correlate with SSA or SSB antibodies in the SS patients (Additional file 1: Figure S1).

### MSC transplantation ameliorates SS-like symptoms in NOD mice

**Fig. 2:**
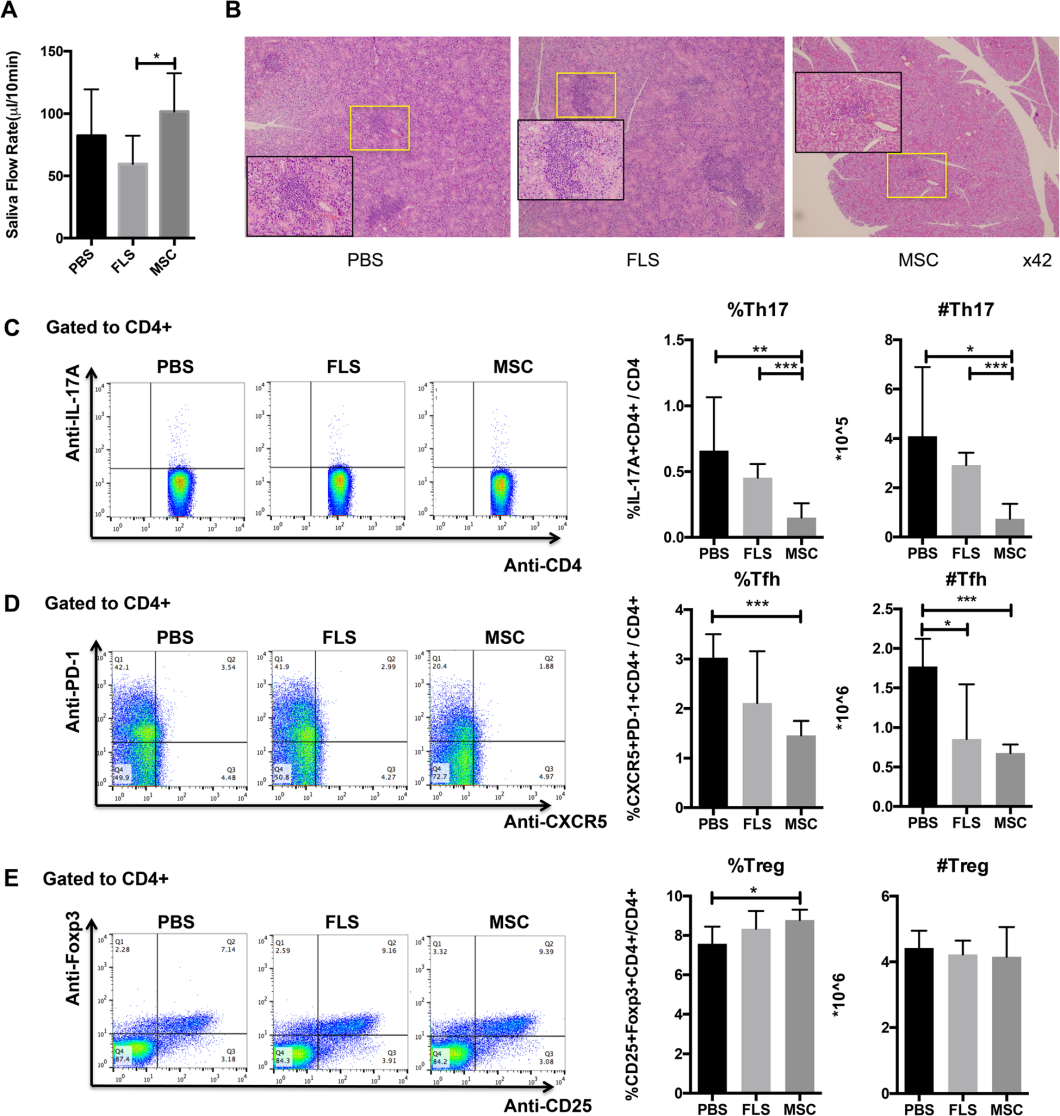
MSCT improved SFRs in NOD mice, ameliorated lymphocytic infiltrations in submandibular glands, and regulated T cell responses. **a** Saliva flow rate of mice at day 28 after MSC/FLS/PBS treatment (*n* = 5 for each group). **b** Histopathology of submandibular glands determined by HE staining (*n* = 5 for each group). **c**–**e** Percentages and absolute numbers of Th17 (CD4+IL-17+), Tfh (CD4+CXCR5+PD-1+), and Treg (CD4+CD25+Foxp3+) cells in splenocytes of mice (*n* = 5 for each group). Data were analyzed by one-way ANOVA. Error bars denote ± SD. *p < 0.05, **p < 0.01, ***p < 0.001

To examine the therapeutic effects of MSCs in NOD mice, we injected 1 × 10^6^ MSCs via tail vein into the mice, with same numbers of FLS or same volume of sterilized PBS as controls. Our results showed a significant improvement of saliva flow rate in the MSC-treated group compared to untreated NOD mice (*p* < 0.05, Fig. 2a). The lymphocytic infiltration in the submandibular glands was decreased in this MSC-treated group (Fig. 2b). The data suggested that MSCs had beneficial roles in improving the salivary gland functions in NOD mice.

### MSC transplantation regulates T cell responses in NOD mice

It was reported that T cell responses, in particular Th17 cells, play critical roles in human pSS and in SS animal models [26, 28]. So we next examined the effects of MSCT on lymphocyte subsets of the NOD mice. MSC transplantation reduced the percentages and absolute numbers of Th17 (*p* < 0.05, Fig. 2c) and Tfh cells (*p* < 0.05, Fig. 2d), whereas upregulated Treg cells in the spleens (*p* < 0.05, Fig. 2e). However, it had no influence on plasma cells (PC), Th1 and Th2 cells (Additional file 1: Figure S2A-C). These results further supported the effectiveness of MSCT in treating SS.

### MSCs inhibit IL-12 production in DCs

**Fig. 3:**
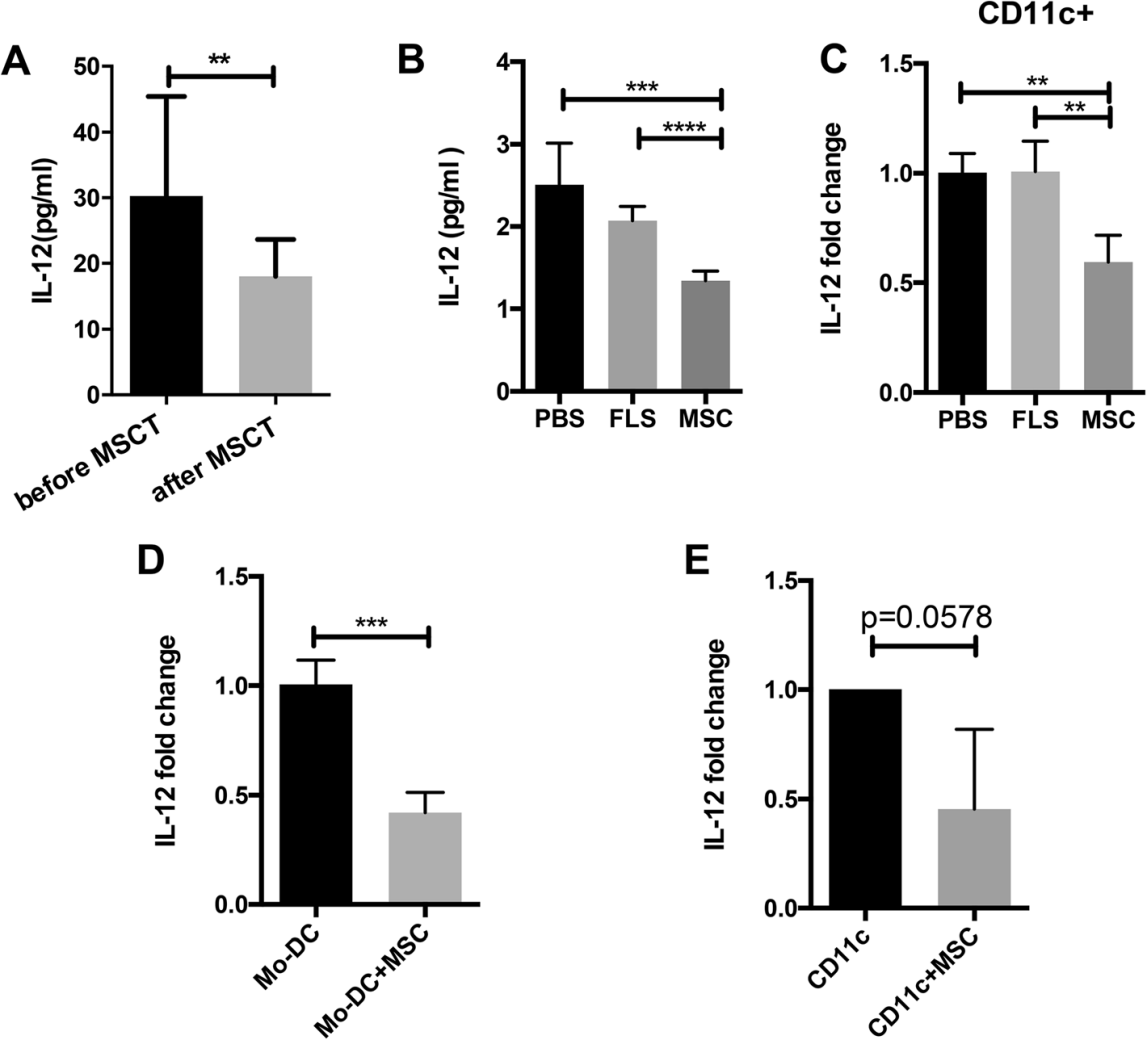
MSCT inhibited human and mouse IL-12 both in vivo and in vitro. **a** Serum IL-12 levels in SS patients before and 7 days after MSCT (*n* = 10). **b** Serum IL-12 levels of mice at day 28 post MSC/FLS/PBS treatment (*n* = 5 for each group). **c** IL-12 mRNA expression in sorted splenic CD11c+ cells 28 days after MSC/FLS/PBS treatment (*n* = 5 for each group). **d** IL-12 mRNA expression in human monocyte-derived DCs cultured alone or co-cultured with MSCs (*n* = 3). **e** IL-12 mRNA expression in sorted splenic CD11c+ cells from C57BL/6 mice cultured alone or co-cultured with MSCs (*n* = 4). Data for **a**, **d**, and **e** are analyzed by paired Student *t* test, and data for **b** and **c** are analyzed by one-way ANOVA. Error bars denote ± SD. **p < 0.01, ***p < 0.001

Next, we detected whether IL-12 levels decrease in parallel with MSCT. In the 10 SS patients enrolled, we found a significant decrease of serum IL-12 levels 7 days after MSCT (*p* < 0.01, Fig. 3a). We then examined IL-12 changes in NOD mice with MSCT or FLS/PBS as controls. We found that 28 days after the treatment, serum IL-12 levels were significantly decreased in the MSCT group (*p* < 0.001, Fig. 3b). Then, we detected IL-12 mRNA expression in the sorted CD11c+ DCs. Notably, CD11c+ DCs in the MSCT group expressed significantly lower levels of IL-12 (*p* < 0.01, Fig. 3c). These data suggest MSCs could inhibit IL-12 productions in vivo, both in the SS patients and mice. We then detected whether MSCs could inhibit IL-12 production in DCs in vitro. Monocyte-derived DCs were used as human DCs; after the co-culture experiment, we found significant inhibited IL-12 expression in the DCs with MSC treatment (*p* < 0.001, Fig. 3d). CD11c+ cells from splenocytes of C57BL/6 mice were used to present mouse DCs; not surprisingly, inhibited IL-12 production was also observed in mouse DCs co-cultured with MSCs (*p* = 0.05, Fig. 3e). These results further supported the findings that MSCs inhibited IL-12 in vivo.

### IL-12 treatment exacerbates disease activity in NOD mice

**Fig. 4:**
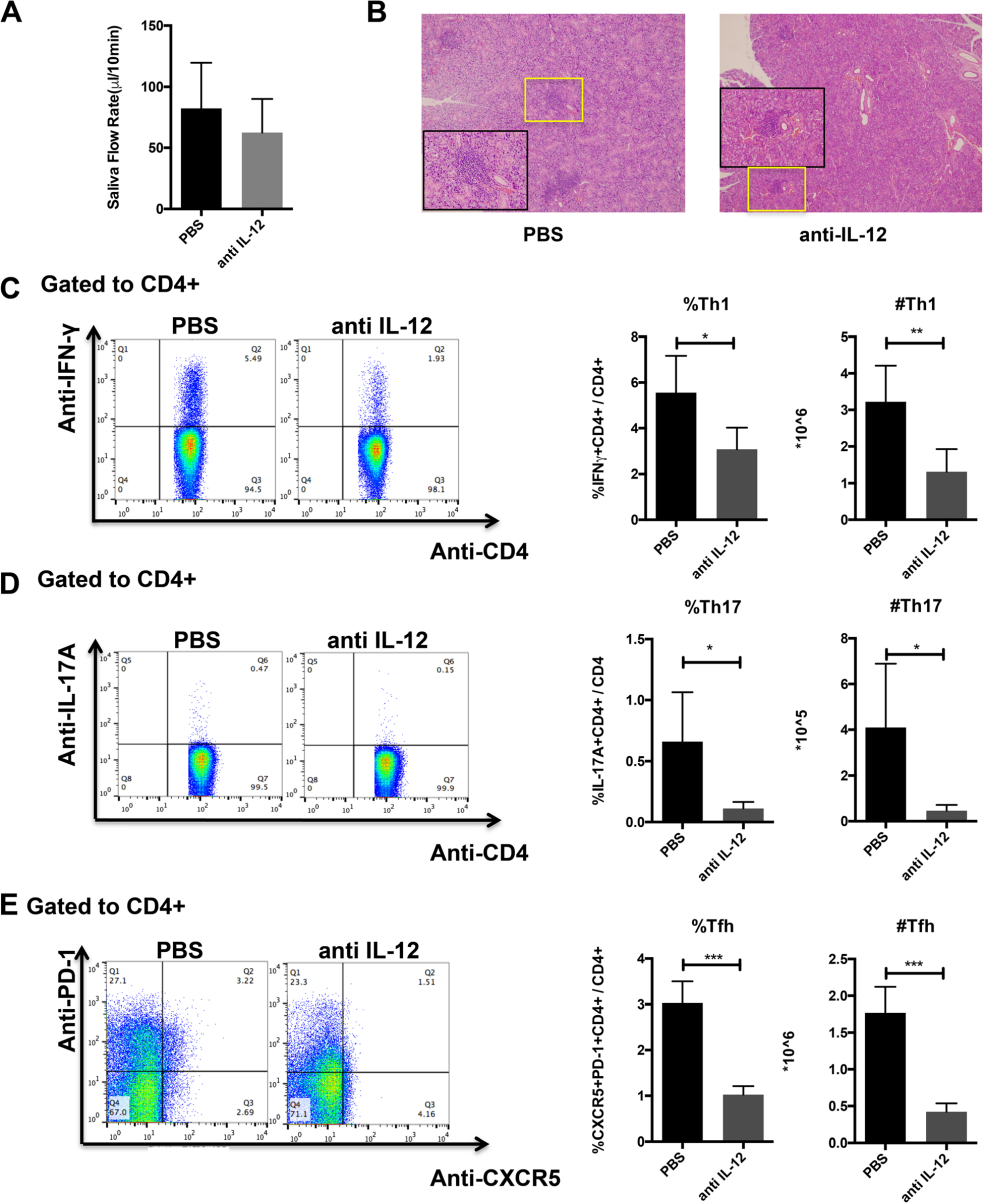
IL-12 antibodies ameliorated SS-like symptoms in NOD mice partially by regulating T cell responses. **a** Saliva flow rates of mice on day 7 after treatment with PBS or IL-12 antibody (*n* = 5 for each group). **b** Histopathology of submandibular glands determined by HE staining (*n* = 5 for each group). **c**–**e** Percentages and absolute numbers of Th1 (CD4+IFN-γ), Th17 (CD4+IL-17+), and Tfh (CD4+CXCR5+PD-1+) cells in splenocytes of mice (*n* = 5 for each group). Data are analyzed by unpaired Student *t* test. Error bars denote ± SD. *p < 0.05, **p < 0.01, ***p < 0.001

We next examined whether in vivo administration of IL-12 or IL-12 antibody could influence the disease progression of NOD mice. Saliva flow rates were comparable between the IL-12 antibody and PBS treatment group (Fig. 4a), although we found milder inflammatory infiltrates in the submandibular glands of IL-12 antibody-treated mice (Fig. 4b). IL-12 antibodies also decreased percentages and absolute numbers of Th1 (*p* < 0.05, Fig. 4c), Th17 cells (*p* < 0.01, Fig. 4d), and Tfh cells (*p* < 0.001, Fig. 4e). No significant differences were found in percentages or numbers of Th2, Treg, and plasma cells (Additional file 1: Figure S3). Collectively, these data demonstrate that IL-12 antibody plays beneficial roles in inhibiting Th1, Th17, and Tfh cells, which were previously reported to be increased in SS patients [26]. On the other hand, recombinant IL-12 significantly decreased salivary flow rates and strongly induced lymphocytic infiltration foci in the submandibular glands, while had weak influence on lymphocyte subsets in the spleen (Additional file 1: Figure S4).

## Discussion

IL-12 had been reported to be increased in several autoimmune diseases. It was elevated in patients with RA and decreased after the patients were treated with disease-modifying anti-rheumatic drugs (DMARD) [29, 30]. Antibodies against IL-12/23 have been proven to be effective in alleviating autoimmune diseases including rheumatoid arthritis, psoriasis, and multiple sclerosis [29, 31, 32].

The role of IL-12 in SS pathogenesis is controversial. Lode et al. reported an increased expression of IL-12 in submandibular glands with severe inflammatory infiltrates in NOD mice, and Ohyama et al. reported higher IL-12 expression in the labial salivary glands of some SS patients [33, 34]. In contrast, Szodory et al. revealed decreased IL-12 levels in SS patients, and in those with extra-exocrine manifestations, IL-12 levels were much lower than those who only suffer from exocrine gland destructions [35]. Here, we demonstrate that IL-12 levels were elevated in SS patients and correlated with disease activity index; our results suggest that IL-12 promoted SS pathogenesis. The reasons for the difference between our and Szodory et al.’s results remain to be further clarified, which at this moment we thought might be attributed to the heterogeneity of the SS patients enrolled. In addition, we did not assort the patients according to the presence or absence of extra-exocrine manifestations, which might be another variant of the results interpreted.

MSCs are multipotent stem cells with strong immune-regulatory functions [36]. MSCs possess immune-regulatory effects on various immune cells like T cells, B cells, DCs, and macrophages [37–40]. MSCs regulate DCs in many aspects. First, MSCs inhibit differentiation of DCs from their precursors, both from monocytes and from CD34+ cells, and this was suggested to be regulated by inhibited cyclin D1 in monocytes and CD1a expression on DCs [41, 42]. MSCs also impair differentiated DCs to express CD80, CD86, CD40, and CD83, which present mature DC phenotype [43]. Moreover, DCs generated in the presence of MSCs were functionally impaired [20]. As IL-12 was mainly produced by activated APCs, especially DCs, we suggest that the inhibition of MSCs on IL-12 might be attributed to the impairment of both DC differentiation and maturation processes.

MSCs lack the expression of co-stimulatory molecules and display low immunogenicity, which make them incapable of alloantigen recognition. Owing to these immunologic features, MSCs have been used in the clinical treatment of autoimmune diseases. Published studies had demonstrated the effectiveness and safety of MSCT for autoimmune disease (AD) [44–46]. We hypothesized that MSCs were effective in treating AD at least in part by regulating cytokine production and the balance of lymphocyte subsets. Indeed, we revealed that MSCs improved the Th17/Treg balance in NOD mice, and inhibited Tfh cells. In addition, we found that IL-12 played a key role in SS partially by inducing saliva loss and regulating T cell responses. Importantly, we uncovered that MSCT reduced IL-12 levels both in patients and animal models, revealing a previously unrecognized mechanism underlying MSC treatment-mediated improvement of Sjögren’s syndrome. However, the molecular mechanisms of how MSCs regulate IL-12 production were not detected in this study, which deserves future investigations.

## Conclusions

In this article, we report that elevated serum IL-12 levels in SS patients correlate positively with disease activity. MSCT ameliorates SS-like symptoms in NOD mice and inhibited human and mouse IL-12 both in vivo and in vitro. Our findings indicate that MSCs ameliorate SS possibly via suppressing IL-12 production and that IL-12 could be a potential therapeutic target of SS.

## Additional file

Additional file 1: **Table S1.** Clinical features of SS patients treated with MSCT. Table S2. Primer sequences for real-time PCR. Figure S1. IL-12 levels did not correlate with SSA or SSB antibodies. Figure S2. A: No inflammatory infiltrates into lacrimal glands in any of the three groups. B-D: MSCT had modest influence on plasma cells (PC), Th1 and Th2 cells. Figure S3. No significant differences were found in percentages or numbers of Th2, Treg and plasma cells in the anti-IL-12 treatment group. Figure S4. IL-12 significantly reduced saliva flow rates and induced lymphocytic infiltrates into submandibular glands in NOD mice, while had modest influence on PC, Th1, Th2, Th17, Treg and Tfh cell subsets in the spleen. (DOCX 1767 kb)

## Abbreviations

AD: Autoimmune disease
ADMSCs: Adipose tissue mesenchymal stem cells
BMMSCs: Bone marrow mesenchymal stem cells
cDNA: Complementary DNA
DCs: Dendritic cells
DMARD: Disease-modifying anti-rheumatic drugs
ELISA: Enzyme-linked immunosorbent assay
ESSDAI: EULAR 2010 Sjögren’s syndrome disease activity index
FLS: Fibroblast-like cells
GM-CSF: Granulocyte–macrophage colony-stimulating factor
HC: Healthy controls
IBD: Inflammatory bowel disease
IL: Interleukin
LPS: Lipopolysaccharides
MSCs: Mesenchymal stem cells
MSCT: MSC transplantation
NOD: Non-obese diabetic
PBMCs: Peripheral blood mononuclear cells
PBS: Phosphate-buffered saline
PGE2: Prostaglandin E2
PMA: Phorbol 12-myristate 13-acetate
RA: Rheumatoid arthritis
SG: Salivary glands
SLE: Systemic lupus erythematosus
SS: Sjögren’s syndrome
UCMSCs: Umbilical cord mesenchymal stem cells
