# Supplementary material for: Mesenchymal stem cell transplantation ameliorates Sjögren’s syndrome via suppressing IL-12 production by dendritic cells
Source: Stem Cell Res Ther. 2018 Nov 8;9:308. doi: 10.1186/s13287-018-1023-x (PMC6225717; doi:10.1186/s13287-018-1023-x)
Supplement: Supplementary file 1 — Table S1. Clinical features of SS patients treated with MSCT. Table S2. Primer sequences for real-time PCR. Figure S1. IL-12 levels did not correlate with SSA or SSB antibodies. Figure S2. A: No inflammatory infiltrates into lacrimal glands in any of the three groups. B-D: MSCT had modest influence on plasma cells (PC), Th1 and Th2 cells. Figure S3. No significant differences were found in percentages or numbers of Th2, Treg and plasma cells in the anti-IL-12 treatment group. Figure S4. IL-12 significantly reduced saliva flow rates and induced lymphocytic infiltrates into submandibular glands in NOD mice, while had modest influence on PC, Th1, Th2, Th17, Treg and Tfh cell subsets in the spleen. (DOCX 1767 kb) [file 13287_2018_1023_MOESM1_ESM.docx]

Table S1 Clinical features of SS patients treated with MSCT

| No. of patient | Age and sex | Disease duration | Organ involvement | Antibody profile | Medications | ESSDAI scores |
| --- | --- | --- | --- | --- | --- | --- |
| 1 | 66F | 10 years | TP, arthralgia | anti-SSA,  anti-SSB | [CsA](file:///F:\Program%20Files\Youdao\Dict\7.2.0.0511\resultui\dict\?keyword=ciclosporin)  (75mg bid) | 9 |
| 2 | 57F | 5 years | hypersplenism, hepatitis | anti-SSA | Pred  (5mg qd) | 5 |
| 3 | 38F | 1 years | TP, erythema | anti-SSA | MMF  (0.75g qd) | 7 |
| 4 | 53F | 5 months | vasculitis, hypothyroidism | anti-SSA | Pred  (25mg qd), HCQ  (0.2g qd) | 5 |
| 5 | 44F | 2 years | arthralgia | anti-SSA, ANA | Aspirin  (100mg qn) | 7 |
| 6 | 53F | 6 years | lymphadenovarix | anti-SSA, ANA | Tripterygium (20mg bid), MTX  (20mg qw) | 9 |
| 7 | 47F | 3 years | HC, leucopenia, arthralgia | anti-SSA,  anti-SSB, ANA | Pred  (15mg qd) | 2 |
| 8 | 60F | 15 years | arthralgia | anti-SSA | HCQ  (0.2g qd) | 4 |
| 9 | 35F | 6 years | TP, erythema | anti-SSA, anti-SSB, | Pred  (80mg qd) | 7 |
| 10 | 50F | 6 months | IN | ANA | Pred  (5mg qd) | 4 |

Note: detailed description of the ESSDAI composites:

1. purpura, hematuresis, cytopenia, hypocomplementemia; 2. fever, cytopenia, cryoglobulinemia; 3. erythema, hematuresis, cytopenia, hypocomplementemia; 4. hematuresis, cytopenia; 5. fever, arthralgia, cytopenia, cryoglobulinemia; 6. hypocomplementemia; 7. lymphadenovarix, arthralgia, hematuresis, hypocomplementemia, cryoglobulinemia; 8. arthralgia, hypocomplementemia; 9. arthralgia, hypocomplementemia; 10. erythema, cytopenia, hypocomplementemia, cryoglobulinemia; 11. hematuresis, hypocomplementemia.

2. Abbreviations: CsA: [ciclosporin](file:///F:\Program%20Files\Youdao\Dict\7.2.0.0511\resultui\dict\?keyword=ciclosporin); HC: hepatic cystic; HCQ: hydroxychloroquine; MMF: mycophenolate mofetil; MTX: methotrexatum; Pred: prednisolone; TP: thrombocytopenia; IN: interstitial nephritis.

Table S2 primer sequences for real-time PCR

| Gene | Primer sequences |
| --- | --- |
| human IL-12(p40) | F: 5’-AGGAATGTTCCCATGCCTTCA-3’  R: 3’-CCAATGGTAAACAGGCCTCCAC-5’ |
| human GAPDH | F: 5’-GCACCGTCAAGGCTGAGAAC-3’  R: 3’-TGGTGAAGACGCCAGTGGA-5’ |
| mouse IL-12(p40) | F: 5’-ACCCTGACCATCACTGTCAA-3’  R: 3’- GTGGAGCAGCAGATGTGAGT-5’ |
| mouse GAPDH | F: 5’-ACAACTTTGGCATTGTGGAA-3’  R: 3’-GATGCAGGGATGATGTTCTG-5’ |


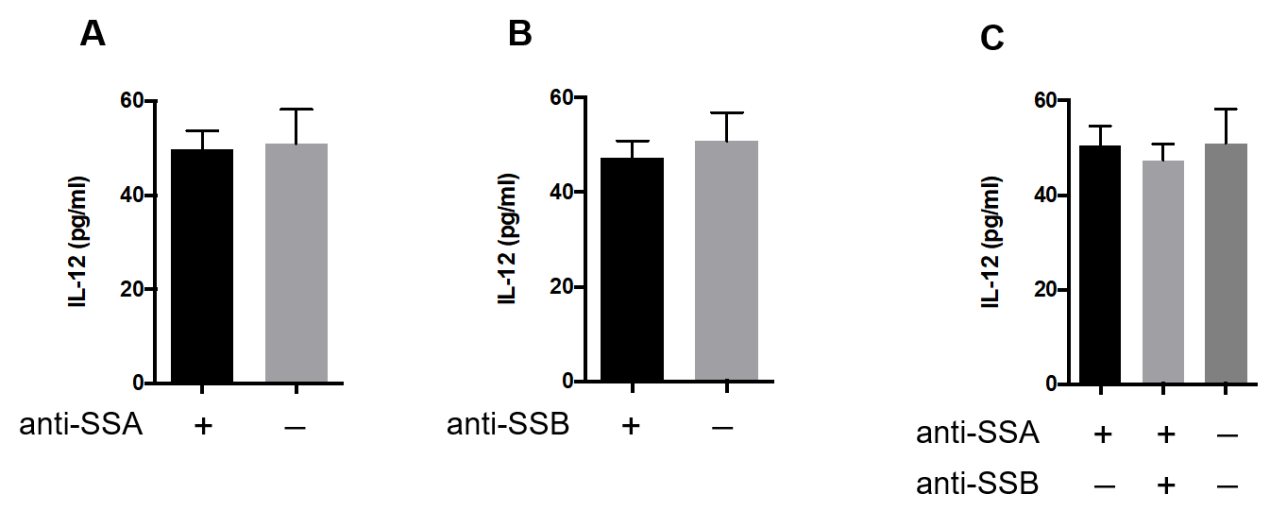
Figure S1: IL-12 levels did not correlate with SSA or SSB antibodies.


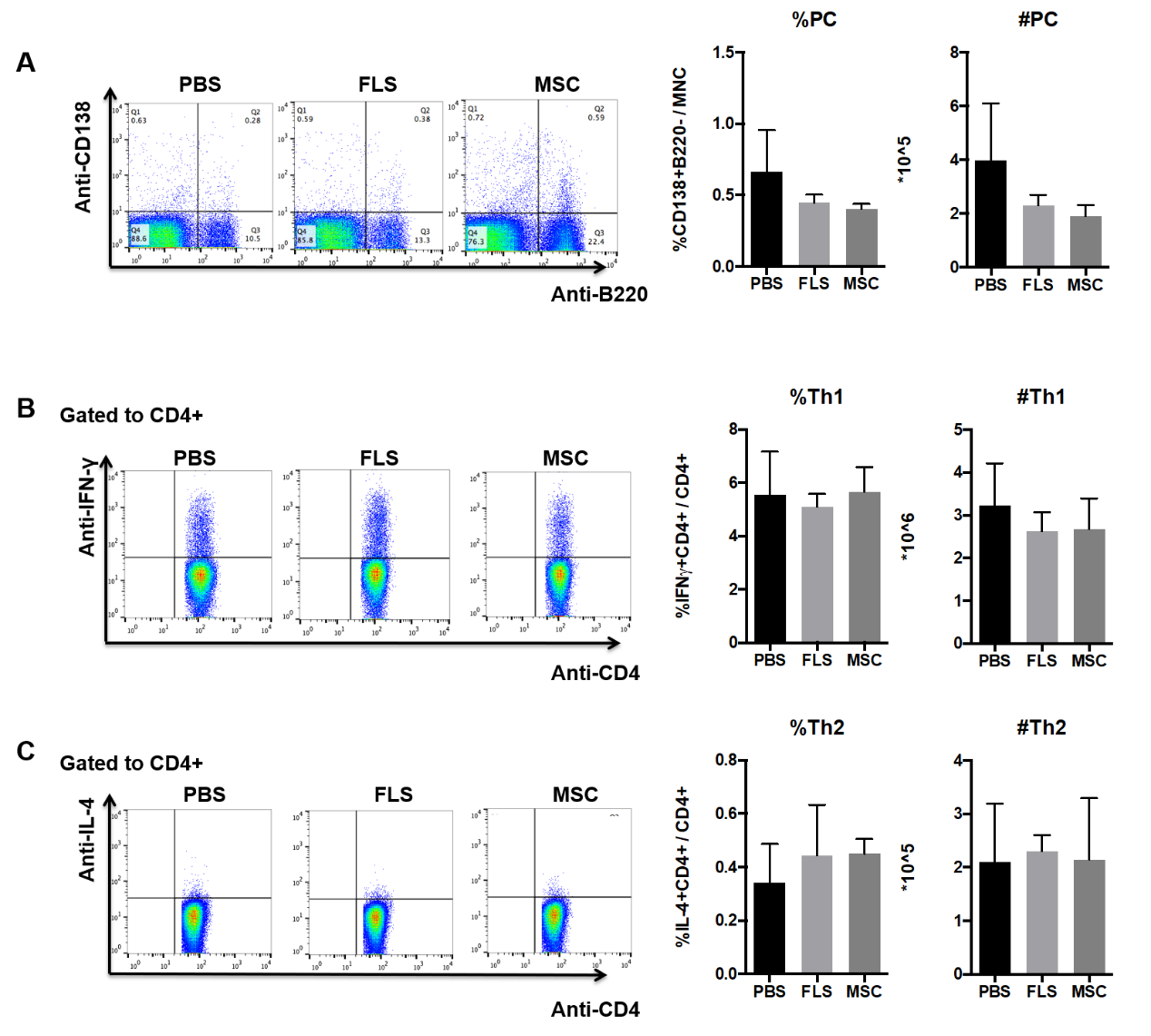
Figure S2: A: No inflammatory infiltrates into lacrimal glands in any of the three groups.

B-D: MSCT had modest influence on plasma cells (PC), Th1 and Th2 cells.


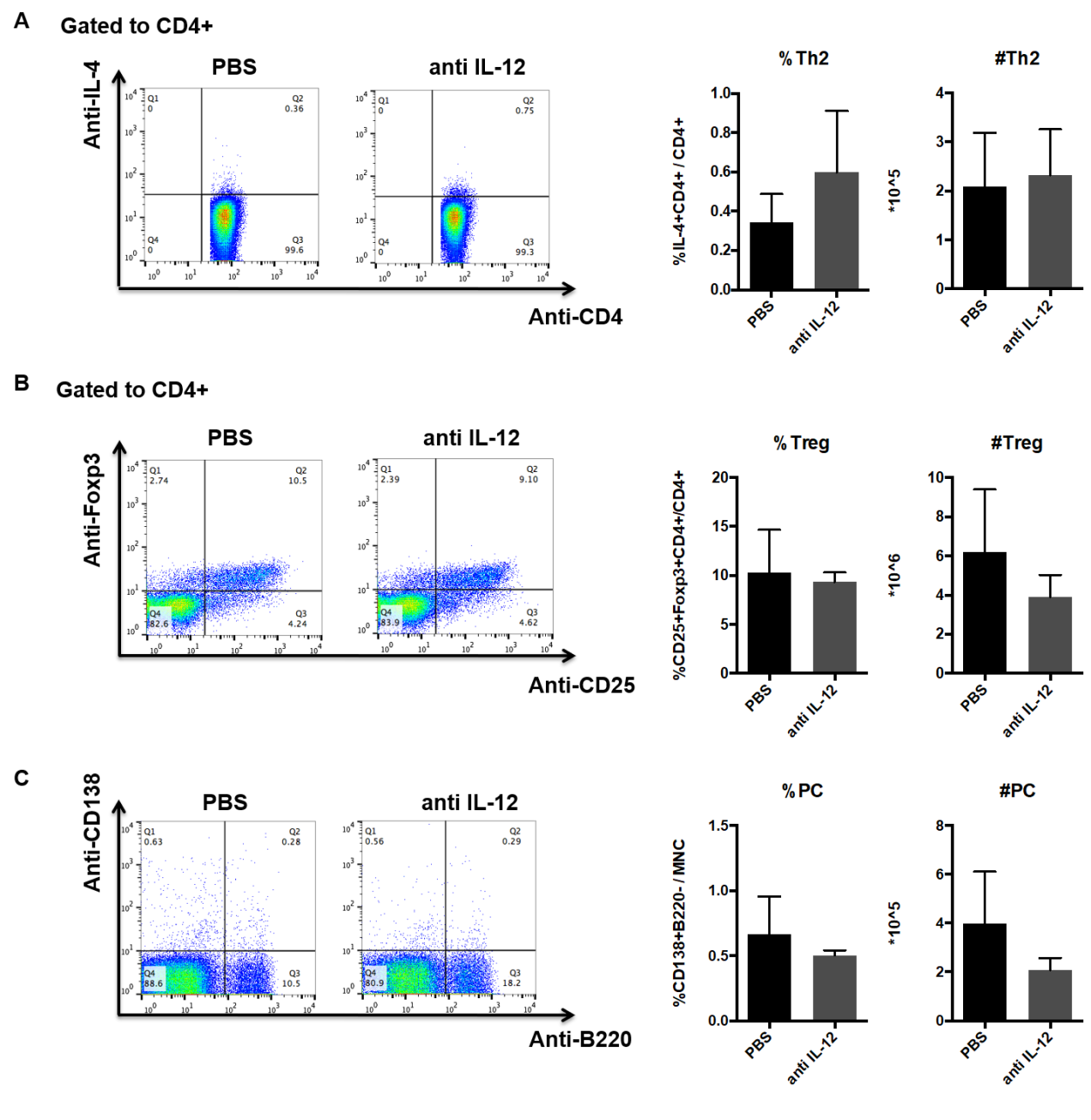
Figure S3: No significant differences were found in percentages or numbers of Th2, Treg and plasma cells in the anti IL-12 treatment group.


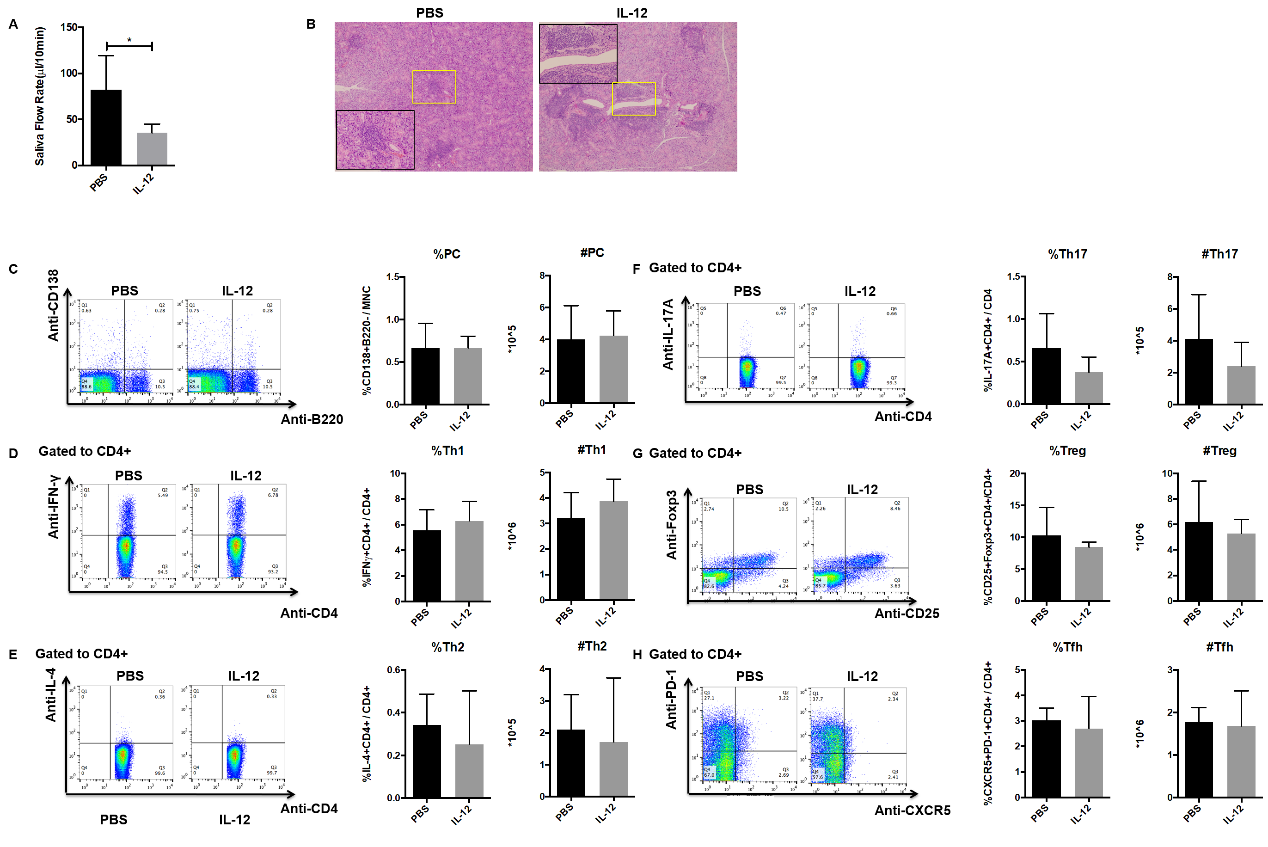
Figure S4: IL-12 significantly reduced saliva flow rates and induced lymphocytic infiltrates into submandibular glands in NOD mice, while had modest influence on PC, Th1, Th2, Th17, Treg and Tfh cell subsets in the spleen.
